# Kinetic analysis of felines landing from different heights

**DOI:** 10.7717/peerj.8007

**Published:** 2019-11-12

**Authors:** Meizi Wang, Yang Song, Stephanie Valentin, Julien S. Baker, Yaodong Gu

**Affiliations:** 1 Faculty of Sports Science, Ningbo University, Ningbo, China; 2 Institute of Clinical Exercise and Health Science, University of the West of Scotland, Hamilton, UK; 3 Department of Sport and Physical Education, Hong Kong Baptist University, Hong Kong, China

**Keywords:** Landing, Motion analysis, Kinetics, Cat, Bionics design

## Abstract

**Background:**

Kinetic motion analysis has been used in canines and equines as a fundamental objective evaluation measurement. Cats are very capable jumpers, and this ability has biomimetic applications. It is essential to understand movement patterns and physical adaptations of this species, as cats are popular pets for humans. Further to this, motion analysis of a cat’s movement patterns may provide potentially valuable information in relation to limb disease and injury. Therefore, the aim of this study was to investigate kinetic differences in cats when landing from varying preselected controlled heights.

**Methods:**

The peak vertical force (PVF) and paw contact area (CA) of both the forelimbs and hindlimbs were collected from seven healthy Chinese domesticated cats while landing from heights of 30 cm, 50 cm, 70 cm and 90 cm respectively. The falling motivation for the cats was facilitated with the use of a flip board. This device provided the basis for the cats to land passively.

**Results:**

The results indicated that the PVF of all examined limbs (fore right, fore left, hind right, hind left) significantly increased as the height increased. When the PVF from the hindlimbs and forelimbs were compared, the forelimbs recorded significantly greater values for all heights examined (*P* < 0.001). The PVF of the hindlimbs was symmetrical at all heights, but forelimb symmetry only occurred at the lower heights. The hindlimbs demonstrated larger CA than the forelimbs measured from all heights on landing (*P* < 0.001). Moreover, the paw CA on the left and right limbs were symmetrical.

**Discussion:**

The paw CA of cats may be an effective parameter to evaluate abnormalities or diseases in the limbs of cats. Additionally, these findings highlight how cats land from varying heights, which may also provide reference values for the bionic design of artificial limbs for felines and treatment for limb diseases in this species.

## Introduction

Cats have a very special body structure that plays a significant role in movements such as running, jumping and landing (Hildebrand, 1959; Koob & Long, 2000). It is a common phenomenon that cats can jump from great heights and land safely, and this has attracted the interest of many researchers. Research related to movement patterns in mammals has been mainly associated with three areas: the patterns of movement (Alexander, Langman & Jayes, 1977; Bertram & Gutmann, 2009), self-stabilization mechanisms (Hackert, Schilling & Fischer, 2006), and the most effective movement mode (Alexander & Jayes, 1981; Alexander, Dimery & Ker, 1985). To date, the last aspect has attracted the most attention. In order to investigate the special landing skills of cats, the self-righting maneuvers of free-falling cats have been captured using high-speed photography. The results demonstrated the crucial role of the cat’s head position in transferring momentum to the body, which facilitated axial rotation allowing the animal to land on its feet (O’Leary & Ravasio, 1984). The cat is considered a hindlimb-driven quadruped, however, the forelimbs appear to have greater force-generating capacity than the hindlimbs (Schnabl-Feichter, Tichy & Bockstahler, 2017). McKinley, Smith & Gregor (1983) attempted to explore the neuronal-control associated with landings in cats from kinematic, kinetic and muscle activity parameters of the elbow joints during forelimb landing following free-falling. In recent years, researchers have investigated the kinetic parameters of PVF and vertical impulse (VI) between the forelimbs and the hindlimbs of jumping cats, and the findings demonstrated that when compared to the hindlimbs, the PVF and VI were significantly larger in the forelimbs of the animals investigated (Stadig & Bergh, 2015). Furthermore, it has been experimentally observed that the PVF of a cat’s contralateral limb (left limb and right limb) were symmetrical during landing from a height of 1 m (Lascelles et al., 2007). However, there have been no investigations examining if the PVF of the cat’s limbs are always symmetrical at different heights.

Studies have demonstrated that kinetic gait analysis can be used to describe normal and disordered locomotion. Data acquisition of gait dynamics provides objective quantification of limb damage, which has been widely applied in both dogs and horses (Rialland et al., 2009).

Interestingly, canines and equines appear to be better able to follow instructions than cats, and adaptation and training responses are important prerequisites for obtaining objective data (Lascelles et al., 2007). This may explain why there are fewer systematic studies published investigating feline kinetics. There is also a need for an effective and intuitive measurement tool that evaluates feline gait and aids in the diagnoses of any potential disease or abnormalities. Osteoarthritis (OA) is a common disease in cats that may cause disability and pain. This disease mainly affects the femoral, stifle and elbow joints, and the prevalence increases significantly with age (Clarke & Bennett, 2006). Most cats with chronic pain caused by OA have no visible signs of lameness (Hardie, Roe & Martin, 2002). In order to explore gait analysis as an effective method of estimating OA prediction, researchers have previously examined 31 healthy cats by using a pressure-sensitive walkway and demonstrated that PVF and VI are reliable gait parameters that can be used to diagnose OA (Lascelles et al., 2006). However, a recent study has demonstrated that compared to VI, PVF was a more reliable diagnostic parameter (Schnabl-Feichter, Tichy & Bockstahler, 2017). Enomoto et al. (2016) found that the values of PVF and VI of walking and landing after jumping from a height of 0.7 m were asymmetrical in the forelimbs of cats that had undergone unilateral onychectomy. In addition to PVF and VI, other parameters of interest include temporal (stride length, stance time and stride time) and ground CA. There are only a few studies that have considered analysis of CA, and previous research has shown that the CA of the right and left limb both in the forelimbs and hindlimbs were equal when the cats were walking (Schnabl-Feichter, Tichy & Bockstahler, 2017).

The physical structure of the cat includes five toes on their front paw and four toes on hind paw (Hongo et al., 1990; Pettersson et al., 1998). Furthermore, the cat’s paw pad plays an important role in daily locomotive activity. The pad comprises a collection of fat and a large number of elastic fibers, which are distributed in the palm and toes of the cat’s foot (Alexander, Bennett & Ker, 1986). The pad can act as a shock absorber and an anti-skid facilitator when the cat jumps from height (Mihai, Alayyash & Goriely, 2015). From a motor control perspective, there is little information available whether it is the full-paw or the middle of the pad providing control when the cat is landing. From a bionics perspective, science, research, and technology can make use of unique biological investigations in relation to the landing ability of cats. This is true not only for robotic bionics, but also in the bionic design of sports shoes using investigation into the cat’s unique landing skill and the structural characteristics of the claw. Previous studies have used ostrich toenails, toe cushions, and metatarsophalangeal joints as biological prototypes to design sports shoes (Liu, 2014).

In order to explore the kinetics of cats during landing, we selected four different heights of 30 cm, 50 cm, 70 cm and 90 cm, respectively, and data was collected from the cat’s limbs during the landing phase. The purpose of the study was to compare any differences between the forelimbs and hindlimbs during the landing phase. Because the forelimbs make the first contact with the ground during landing, our research questions focused on two basic areas: (1) How does the PVF of each limb change at different heights? (2) How does the paw CAs of the forelimbs and hindlimbs change after landing from different heights?

## Materials and Methods

### Animals

Seven healthy Chinese domesticated cats aged between one and five years were recruited via written consent from their owners for voluntary participation in the study. Prior to data collection, each cat was given a full clinical examination to ensure that all the animals were free from orthopedic and neurological disease and injury. This examination included X-ray of the hips, stifles and elbows. All seven cats were healthy, three were female and four were male, with a mean body mass of 4.0 ± 0.8 kg, the details are listed in Table 1. The body condition score of the cats was based on the criteria proposed by Laflamme (1997). The study was approved by the Animal Ethics Committee of Ningbo University (NBUAEC20170612).

**Table 1:** The details for seven cats.

Number	Gender	Age (year)	Masse (kg)	Body condition (score)
1	Male	2.3	3.8	5
2	Male	3	4	5
3	Male	4.5	4.8	7
4	Male	1.7	3.2	5
5	Female	3.4	4.3	6
6	Female	2.8	4	5
7	Female	2	3.6	5

### Equipment and experimental design

Dynamics data was collected using a pressure sensing mat EMED-AT system (Novel, Germany), the size was 700 × 403 × 15.5 mm and had a sensor area of 475 × 320 mm, which contained up to 6080 sensors with a recording frequency of 100 HZ. The pressure sensing mat was interfaced to a computer and used EMED data analysis software to capture and analyze the data. Previous research studies that investigated kinetics in cats, have used a pressure sensitive walkway. There is limited data in the literature describing the use of pressure sensing mats to collect kinetic data on cats (Schnabl & Bockstahler, 2015). However, in spite of this, researchers have indicated that pressure sensing mats can provide more comprehensive and accurate data than other devices. Pressure sensing mats also allow for estimates of vertical, mediolateral and craniocaudal forces. These forces can be captured at simultaneously, and the high and low pressures generated can be evaluated accurately (Demes et al., 1994; Corbee et al., 2014).

It is difficult for cats to follow experimental instructions, even when using food or toys to manipulate attention. Thus, in order to keep the experiment running smoothly, a lifting flip board was used to encourage the animals to fall consciously in a safe manner. To ensure the safety of the cats in the experiment and to be able to get exclusive data, the height of the table was set at 30 cm, 50 cm, 70 cm and 90 cm respectively.

**Figure 1:**
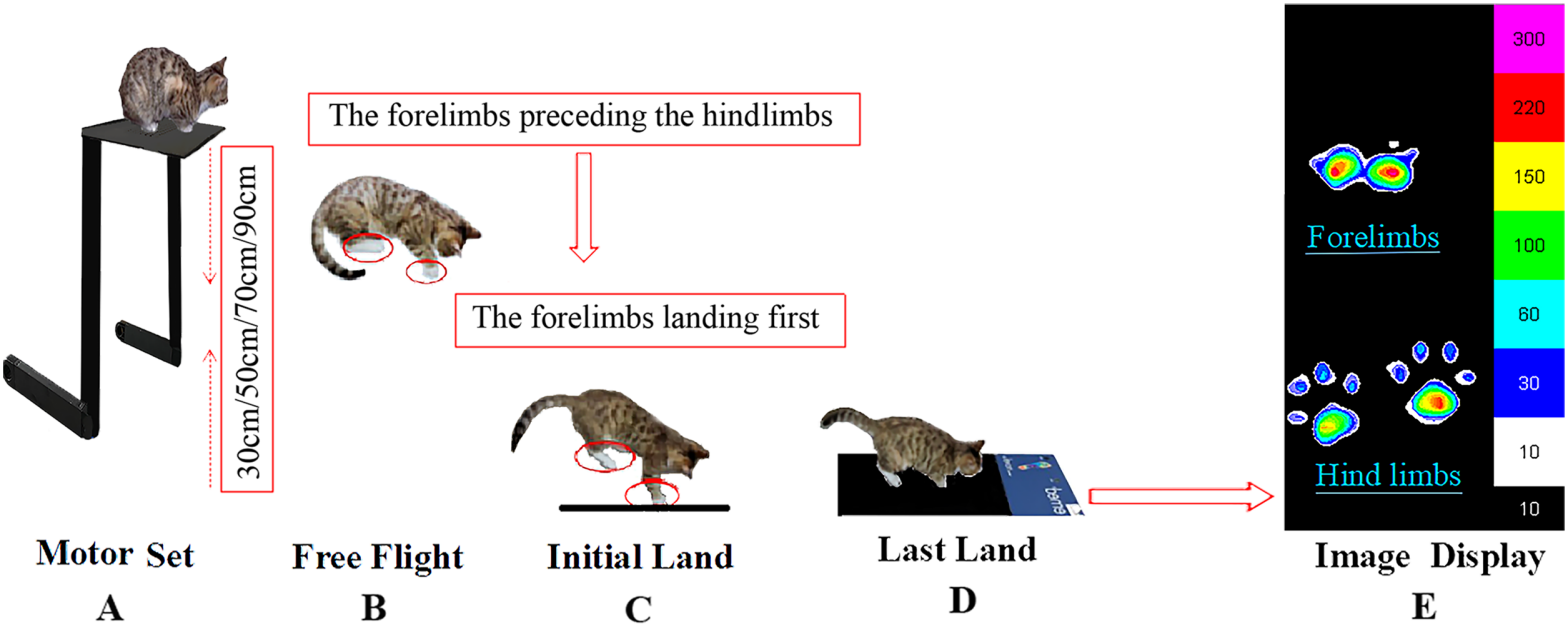
The experimental view of the cat falling from the board to the pressure sensing mat from different heights. (A) Motor set, (B) free light, (C) initial land, (D) last land, (E) image display.

The study was conducted in collaboration with the cat owners, the cats were kept in a quiet state and comfortable environment in the lab. In the initial phases of experiment, the cats were nervous about the unfamiliar environment, and the experimenters relaxed the animals with toys and food. When the cats were deemed to be calm, they were included in the experiment with their owners present throughout the data collection period. The cat owners encouraged the cats to sit in a squat position on the experimental table while the height of the table was adjusted to the specifically required height. There was no apparent tilt of the body, and the cat’s head and body were facing forward when the cat landed. Prior to experimental data collection, the cats had several adaptation trials to familiarize them with experimental procedures and conditions. To avoid fatigue, each cat only performed one height per day. Each cat was tested five times per height to ensure validity and reduce experimental data collection error. A fall was judged successful when the cat’s limbs all landed on the pressure sensing mat, and when the animals could continue walking forward. When these conditions were observed, the data was deemed reliable and captured for future analysis. None of the animals were injured or suffered adverse effects following participation in the experiment. The ready position, take-off and landing phase of individual falls from different heights is illustrated in Fig 1.

### Data analysis

Prior to the experiment, a power of the test analysis was conducted. It was concluded that a sample size of seven animals was required for experimental integrity and the power value recorded was 0.95. The kinetic parameters of the four paws were collected and the PVF of the four limbs were compared for different fall heights. The PVF and paws CA for the forelimbs and hindlimbs for the different heights were also compared. The PVF value for each cat was normalized to body weight (Stadig & Bergh, 2015). Statistical analysis was performed using statistical software SPSS 19.0 (SPSS Inc, Chicago, IL, USA). In order to assess any differences in kinetic parameters, independent sample *t*-tests and one-way ANOVA were used in conjunction with Bonferroni corrections. For all analyses, the significance level was set at 0.05. Prior to statistical analysis, the homogeneity test of variance was performed on the data, and the test results showed that homogeneity of variance was established. The normality of the data distribution was also examined and the results showed that the data were normally distributed.

## Results

All cats completed the acquisition of kinetic data. The mean PVF and paw CA from the four measurement heights are shown in Tables 2 and 3. All tested limbs (fore right, fore left, hind right, and hind left) in the different heights were significantly different. Compared to the 30 cm, the PVF of the fore right following the 50 cm fall showed a significant increase (30 cm vs 50 cm; *P* < 0.001). The 70 cm fall produced a significantly larger PVF compared to the 50 cm (50 cm vs 70 cm; *P* = 0.008). Compared with the 70 cm, the 90 cm fall produced the greater PVF (70 cm vs 90 cm; *P* < 0.001). The PVF of the four limbs all showed a similar increasing trend with the increase in fall height.

**Table 2:** The peak vertical force (PVF, %BW) value of each limb of the cat was compared between right limbs and left limbs.

	30 (cm) Mean ± SD	50 (cm) Mean ± SD	70 (cm) Mean ± SD	90 (cm) Mean ± SD
Fore, right	76.51 ± 5.12	104.51 ± 9.23	117.47 ± 8.95	149.74 ± 9.57
Fore, left	75.47 ± 7.83	94.64 ± 9.05	106.11 ± 7.04	125.77 ± 8.09
Minimal detectable difference	13.04	14.93	15.97	19.67
P (FR vs FL)	0.7	0.06	0.02	<0.001
Hind, right	60.79 ± 1.70	82.56 ± 2.5	98.81 ± 9.11	120.21 ± 7.66
Hind, left	62.18 ± 1.53	83.77 ± 2.54	103.57 ± 8.64	117.92 ± 2.54
Minimal detectable difference	11.73	13.64	14.45	17.01
P (HR vs HL)	0.13	0.38	0.33	0.48

**Table 3:** The mean value of paw contact area (CA, cm^2^) of all limbs in different heights.

	30 (cm)	50 (cm)	70 (cm)	90 (cm)
Fore, right	2.87 ± 0.39	3.09 ± 0.44	3.3 ± 0.2	3.6 ± 0.47
Fore, left	2.84 ± 0.49	3.03 ± 0.55	3.36 ± 0.4	3.7 ± 0.59
Minimal detectable difference	2.2	2.62	2.71	2.85
P (FR vs FL)	0.9	0.83	0.74	0.74
Hind, right	3.6 ± 0.57	4 ± 0.56	4.87 ± 0.57	4.83 ± 0.26
Hind, left	4.07 ± 0.52	4.26 ± 0.61	5.10 ± 0.22	4.81 ± 0.7
Minimal detectable difference	2.9	3.01	3.33	3.26
P (HR vs HL)	0.13	0.42	0.34	0.97
Forelimbs	5.71 ± 0.83	6.11 ± 0.85	6.65 ± 0.43	7.3 ± 0.81
Hindlimbs	7.67 ± 0.53	8.25 ± 0.83	9.97 ± 0.67	9.64 ± 0.87
Minimal detectable difference	3.85	3.99	4.3	4.35
P (FLS vs HLS)	0.001	<0.001	<0.001	<0.001

**Note:**

The force values of the anterior and posterior limbs, contralateral limbs are compared. FR, fore right; FL, fore left; HR, hind right, HL, hind left; FLS, forelimbs; HLS, hindlimbs.

**Figure 2:**
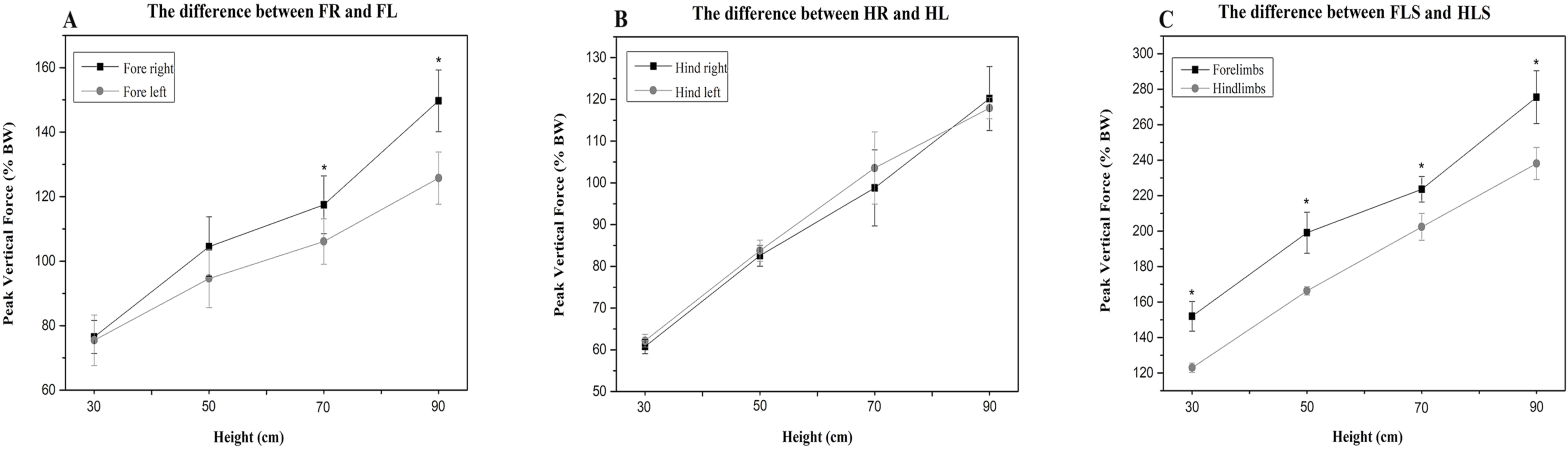
The PVF of the cats’ limbs during landing from different heights. (A) the difference between FR and FL; (B) the difference between HR and HL; (C) the difference between FLS and HLS. The symbol “*” represents a significant difference (*P* < 0.05). FR, fore right; FL, fore left; HR, hind right; HL, hind left; FLS, forelimbs; HLS, hindlimbs.

**Figure 3:**
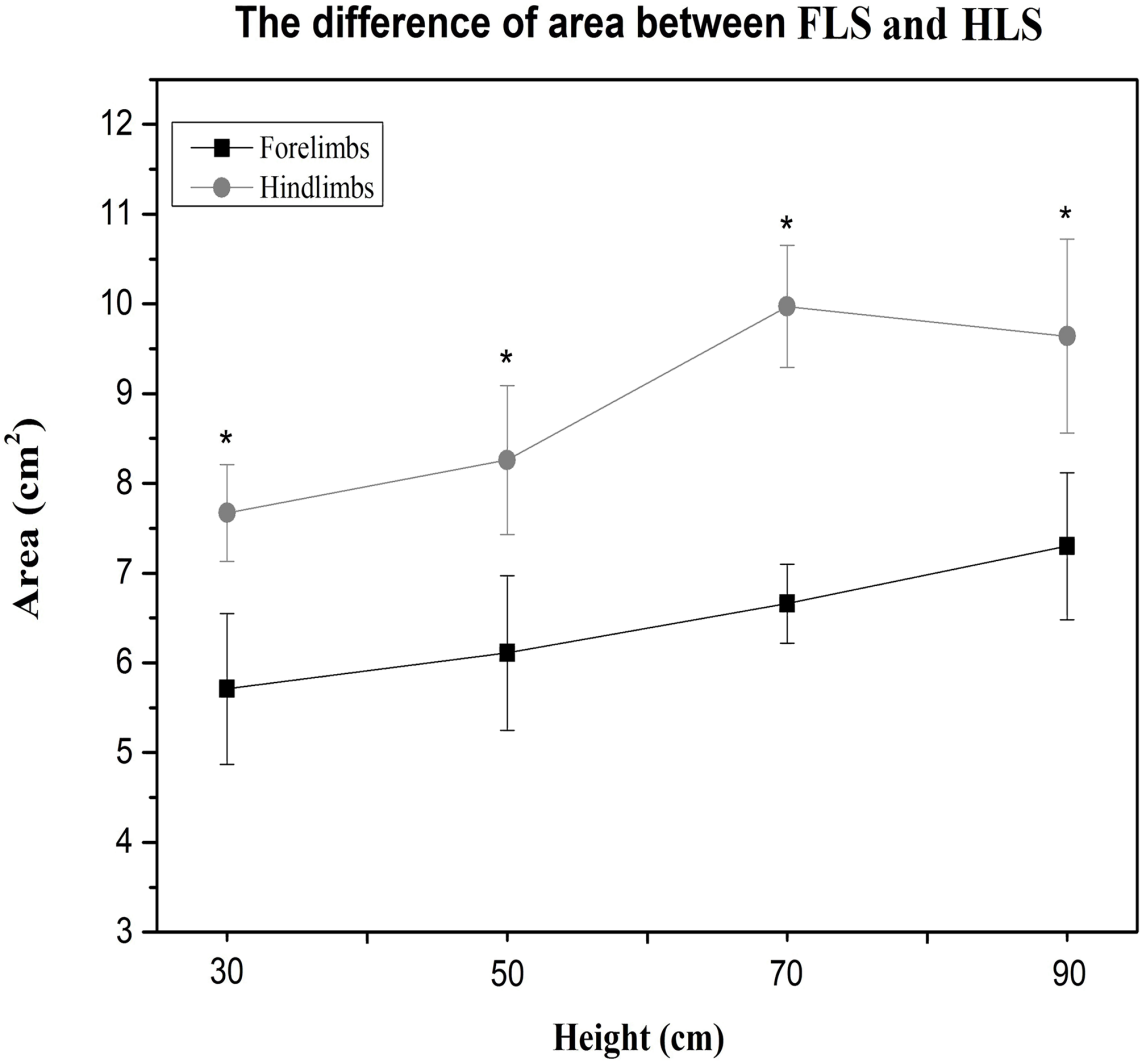
The paw CA of cats between the forelimbs and hindlimbs in different heights; the CA of the forelimbs is significantly larger than the hindlimbs (*P* < 0.05).

Compared to the hindlimbs, the PVF of the forelimbs showed significantly greater values for 30 cm, 50 cm, 70 cm and 90 cm (student’s *t*-test, *n* = 7, *P* < 0.001). A significant difference for PVF was found between the fore right and fore left during the 70 cm (student’s *t*-test, *n* = 7, *P* < 0.05) and 90 cm fall (*P* < 0.05), the fore right was significantly larger than the fore left (Fig. 2A). There were no differences in CA between right and left limbs at all heights (Table 3). Compared with the forelimbs, the hindlimbs demonstrated a significantly greater CA at all heights (student’s *t*-test, *n* = 7, *P* < 0.001) (see Fig. 3).

## Discussion

The motto “A cat has nine lives” highlights a cat’s excellent survivability from falls. In order to examine how cats land, this study measured the variation in PVF and the CA of foot pad in Chinese domesticated cats during landing from different heights using a pressure sensing mat. The findings from this study indicate that the PVF changes in all limbs as a result of landing from different heights. Obviously, in this study as the height increased from 30 cm to 90 cm, the PVF on landing increased in the four limbs respectively. The PVF of the forelimbs was greater than the hindlimbs at any height which confirms the cats are forelimb dominant. When the cats were jumping, the elbow of the forefoot extended, the center of gravity of the body moved forward, and landing posture was constantly adjusted until it gradually shifted to the forelimbs, then the elbow joint continuously extended and reached maximum extension for initial landing of the forelimbs (McKinley, Smith & Gregor, 1983). Previous studies have demonstrated that the function of the cat’s limbs acts as a “mechanical buffer” during landing, which is based on both neural control and muscle tendon unit control functions (Santello, 2005; Prochazka et al., 1977). During the passive fall, the cat may experience minimal reaction time, therefore, the muscle control function of the forelimbs may play a role during landing, especially from low heights, such as 30 cm and 50 cm. The antebrachium and forelimb muscles of the elbow region are thought to be suitable for absorbing the impact, as they have long tendons and muscle fascicule (English, 1978). In particular, the long tendons, which act as power attenuators protect the forelimb muscles from any damage caused by rapid and forceful prolongation during power dissipation at landing (Roberts & Azizi, 2010; Konow, Azizi & Roberts, 2012). Thus, it seems that the forelimbs can effectively dissipate impact under the control of the cat’s muscle and tendon.

In this study, the symmetry of PVF from different heights was also determined, and the results showed significant differences between the fore right and fore left at 70 cm and 90 cm respectively, as outlined in Fig. 2A. According to previous research, cats jumping from a meter in height landed simultaneously and the forces on the left and right sides were equal (Stadig & Bergh, 2015). Moreover, Corbee et al. (2014) evaluated that there was 97% symmetry in the PVF between the contralateral limbs in walking. The equal value of PVF and contact time between the right and left limbs during landing can be used as an additional indicator to diagnose orthopedic symptoms in cats (Schnabl-Feichter, Tichy & Bockstahler, 2017). The findings from this study indicated that only the hind paws in relation to PVF were symmetrical at different heights (Fig. 2B). In the forelimbs, the symmetry of PVF was observed only at the lower heights of 30 cm and 50 cm, and the difference in PVF between fore right and fore left was greater when height increased. This result was inconsistent with previous studies that indicated that the PVF of the cat’s forelimbs was always symmetrical when jumping from different heights. Compared with previous research, this result may be due to the different cat tail positions presented by different drop modes. In this experiment, the cat was passively dropped. During this process, the cat’s tail was not elevated, and related research has proved that the tail of the cat can maintain body balance during movement. Therefore, the asymmetry of forelimb PVF may be the result of self-balancing regulation influenced by the effect of the cat’s loss of tail regulation. It is also worth noting that cats have the initiative to adjust their body posture and achieve optimal allocation, and can adjust the landing angle as the height increases. Moreover, the muscular activity of the cats may be influenced by experimental conditions (McKinley & Smith, 1983). For example, the timing and magnitude of the forelimb muscle activity before landing will be adjusted according to the landing height by the cat (Demes et al., 1994). As described above, in this experiment, the cats subjected to a higher height had sufficient response time to adjust, and the PVF asymmetry of the forefoot was probably the optimal allocation for the cat to achieve a stable landing. Thus, the PVF symmetry of the contralateral limb as an indicator of cat OA needs to be further verified in future studies.

An interesting finding from this study was that the CA of the cat’s paws was significantly different between the forelimbs and hindlimbs. The CA of the forelimbs was always less than the hindlimbs with only the central pad in contact with the ground at initial landing. This can be observed from the image of the CA distribution of the collected claw pads (Fig. 1). The results demonstrate that the central pads of the forelimbs play a major role in ground support during the first contact in the landing phase. From the perspective of neural control functions, the central pad was probably evoked by central lobes which can deliver the strongest excitement signal, and the toe muscles were controlled by the medial and lateral lobes where the stimulus signal is usually asymmetrical and weaker (Hongo et al., 1990). Furthermore, the plantar stimulation in the central pad is greater with increased activation to the limb muscles. In contrast, the plantar stimuli were applied to the toe pads without causing any large movements of the toes or other proximal joints. It appears that the central pad played an important role in the stability of the limbs (Hongo et al., 1990). This finding may be used as an additional indicator to evaluate different pathologies and to assess treatment responses in felines.

Limitations of this experiment included the difficulty in encouraging the cats to fall on to the pressure sensing mat from the table. Previous studies employed food or toys to tempt the animals to jump. However, this approach was unsuccessful in this experiment, so a passive system was developed to encourage the animals to participate. In addition, further research should consider the different breeds of cats. This is the first study to examine the Chinese domesticated cat and European breeds of cats have been more commonly used in previous studies. There are also differences in motion performance with different breeds of cats, so there needs to be a consistent approach. In order to investigate further OA development in cats, and to provide a comprehensive basis for the development of modern bionics, further studies should consider the kinetic differences of different breeds of cats providing specificity in answers to specific research questions.

## Conclusions

This study provides valuable information on the landing ability of felines from different heights by utilizing a pressure sensing mat for data capture. The PVF of the forelimbs was greater than the hindlimbs. As the height increased, the PVF of both the forelimbs and hindlimbs significantly increased. The PVF of hind right and left were symmetrical at different heights, but the PVF symmetry of the contralateral forelimbs was only observed in the 30 cm and 50 cm heights. Compared to the hindlimbs, the paw CA of cats in the forelimbs was always smaller than hindlimbs at any height. The foot pad in the forelimbs was also shown to play a major role during landing. In addition, the CA on the left and right sides of the cat were found to be symmetrical. From the perspective of bionics, research on the unique skill the cat has for landing provides reference, inspiration and potential further research for bionic design and/or bionic movement.

## Supplemental Information

10.7717/peerj.8007/supp-1 Supplemental Information 1 The data of paw contact area (PCA, cm^2^) in different heights.

10.7717/peerj.8007/supp-2 Supplemental Information 2 The data of peak vertical force (PVF，%BW) in different heights.

10.7717/peerj.8007/supp-3 Supplemental Information 3 Video of Cat Testing.

## Additional Information and Declarations

### Data Availability

The following information was supplied regarding data availability: The raw data is available as Supplemental Files.
